# Universal Health Coverage in Pakistan: Challenges, Reforms, and the Road to 2030

**DOI:** 10.5334/aogh.5238

**Published:** 2026-08-31

**Authors:** Babar Tasneem Shaikh

**Affiliations:** 1 School of Public Health, Health Services Academy, Park Road, Chak Shahzad, Islamabad 44000, Pakistan

**Keywords:** universal health coverage, health system strengthening, primary health care, health financing, Pakistan

## Abstract

*Background:* Universal Health Coverage (UHC) aims to ensure that all individuals have access to essential health services without financial hardship. Pakistan, despite constitutional commitments and policy reforms, faces significant hurdles in progressing toward this global goal due to systemic, structural, and socioeconomic challenges.

*Methods:* This narrative review synthesizes available literature, policy documents, and programmatic evidence to examine Pakistan’s preparedness for UHC. The analysis is structured around four thematic levels: policy, institutional, population, and service delivery, identifying barriers and opportunities within each domain.

*Findings:* At the policy level, political instability, inadequate financing (<1% of GDP), and lack of evidence-based decision-making undermine sustained progress. Although a comprehensive UHC benefit package has been developed, its implementation is constrained by insufficient budget allocations. Institutionally weak regulation, fragmented governance, inequitable distribution of human resources, and limited stakeholder coordination impede service delivery. At the population level, low health literacy, poor awareness of insurance entitlements, and socioeconomic disparities result in low utilization of available services. Service delivery challenges include questionable quality of care, weak hospital empanelment standards, absence of standardized treatment protocols, and poor communication between public and private sectors. Out-of-pocket expenditures remain catastrophically high, pushing vulnerable populations further into poverty.

*Conclusion:* Pakistan’s path to UHC by 2030 requires a fundamental paradigm shift. Priorities include increasing health financing to at least 3% of GDP, strengthening primary healthcare as the platform for equity, scaling up social health protection programs nationwide, investing in workforce development and digital health infrastructure, and launching contextualized public awareness campaigns to improve health insurance literacy. A multi-sectoral, governance-focused approach is essential to translate policy intent into tangible health outcomes for all citizens.

## Background

Universal Health Coverage (UHC) is the next crucial milestone for the global community, which will ensure that all individuals have access to essential health services without facing financial hardship [1]. Reaching UHC would necessitate a strong and inclusive health insurance system. Pakistan has a large portion of the population without financial protection against the high costs of medical care [2]. This state of affairs forces millions of individuals and families to bear the expenses out-of-pocket, often pushing them into poverty or altogether preventing them from seeking necessary healthcare services [3]. In spite of some notable efforts, the country faces several challenges *en route* to UHC, which are deeply rooted in systemic, structural, and socioeconomic issues [4]. Ambitious benefit package design, weak monitoring, low health insurance literacy and low utilization of services, a dearth of skilled human resources, questionable quality of care and standards, varying empanelment criteria for hospitals, inadequate health financing, and the overarching political unsteadiness in the country [5].

This narrative review was informed by a targeted search of the published and grey literature. Electronic databases, including PubMed, Scopus, and Google Scholar, were searched for English-language publications from January 2010 to March 2025 using combinations of keywords related to *universal health coverage, health systems, health financing, primary healthcare, health insurance, governance*, and *Pakistan*. Additional evidence was identified from reports and policy documents published by the Government of Pakistan, the World Health Organization, the World Bank, and other international agencies. Publications were selected based on their relevance to Pakistan’s progress toward UHC, with priority given to peer-reviewed studies, national policy documents, and reports addressing health financing, governance, service delivery, human resources for health, and health system performance. As a narrative review, the objective was to provide a comprehensive synthesis of the available evidence rather than a systematic appraisal of study quality.

## Policy Level

### Political instability and governance issues

Frequent changes of governments and their respective mandates upset the continuity of health policies, agendas, and programs [1, 6]. This directly affects the anticipated outcomes of the programs designed for the welfare of the underserved segments of the population. Furthermore, mismanagement of the public funds may undermine the initiatives geared toward improvement of the health system infrastructure, workforce, and service delivery [7].

### Legislation

Legal frameworks are also needed to operationalize the programs for achieving UHC, ensuring equity, quality, and safety of the users of health services and for protection of their rights. Such a framework would clarify the terms of reference for health workers, health agencies, and health systems, and establish legal and regulatory procedures to confirm that health systems and the relevant stakeholders are accountable [8]. Pakistan, as a resource-constrained country, is yet to develop effective mechanisms and a policy direction for a fair distribution of resources so that vulnerable populations are safeguarded, and health inequalities are addressed.

### Healthcare financing and resource allocation

Insufficient healthcare financing would emerge as one of the barriers to achieving UHC in Pakistan, a country that allocates less than 1% of its GDP to healthcare, notably lower than the global average [9]. This meager funding allocation has resulted in inadequate infrastructure, shortage of supplies, and insufficient health coverage, specifically in rural and remote parts of the country. Many government hospitals do not have enough stocks of essential medicines, medical equipment is outdated, and the quality of care is highly questionable [10]. People are spending grossly out-of-pocket for purchasing quality healthcare in Pakistan, accounting for as much as 53% of total healthcare costs [11]. This high out-of-pocket cost becomes a major deterrent to seeking appropriate needed medical care [12]. Although a health insurance program that does not cover the entire poor population, many people who deserve to be covered by this safety net continue to suffer due to catastrophic health expenditure, a major determinant of poverty [13].

### Evidence-based decisions

Evidence-based decision-making can facilitate the development of health policies and programs that are well backed up by credible research [14]. Use of evidence can inform decision-makers on efficient resource allocation and help develop programs that could cater specific needs of the larger population [15]. Moreover, understanding the sociocultural determinants and community norms helps in aligning the interventions according to the health needs and services [16]. The literature suggests that strengthening evidence-informed policymaking could support progress toward UHC.

### Benefit package

Globally, Pakistan is one of the leading countries to have adopted the Disease Control Priorities 3 (DCP3) evidence and developed a set of interventions for shaping the content of the benefit package [17]. The benefit package was based on a well-thought-out governance and institutional arrangement, as well as an evidence valuation with cost analysis. The district-level package comprises 117 interventions, of which 88 can be immediately implemented (@US$12.98), and the remaining will have to wait until the health budget is increased to meet the costs of the full package (@US$29.70). Once fully implemented, this benefit package is likely to forestall up to 46.75 million Disability Adjusted Life Years (DALYs) approximately [18]. These findings suggest that increasing health budget allocation may be necessary to ensure allocative efficiency in order to fully achieve the ambitious targets of UHC.

## Institutional Level

### Monitoring

To monitor the composite progress of UHC in all countries, tracer indicators were defined [19]. These tracer indicators were selected for the index, including four from each of the categories of reproductive, maternal, newborn, and child health; infectious disease; and three each from the categories of noncommunicable diseases and service capacity and access. According to recent data, Pakistan is lagging behind on women and child health indicators and is unlikely to achieve the targets set by the Sustainable Development Goals [20]. Furthermore, monitoring the progress toward UHC is becoming difficult in the absence of a national-level survey because of a dearth of funds from development partners.

### Regulation

There have been many challenges regarding the regulation of the health system in Pakistan. A lack of robust regulatory measures and machinery adversely affects equitable access to healthcare, financial protection, and quality of service delivery. Weak federal–provincial coordination, lack of capacity to implement health sector strategies, and a frail oversight mechanism have been associated with an undependable service delivery [21]. There is a massive private sector that is largely unregulated and unlicensed, and many clinics and hospitals function without necessary quality checks. Free private health market with no standard prices results in the exploitation of patients, particularly those who are non-affording. Essential medicines are often expensive or inaccessible. Substandard and counterfeit medicines are sold without any fear. Equipment quality and safety are also questionable [22].

### Insufficient and inefficient human resources

Pakistan faces a serious shortage of nurses and paramedical staff [23]. Moreover, a significant number of skilled healthcare workers prefer to migrate abroad for greener pastures, hence causing brain drain [24]. The inequitable distribution and deployment of health personnel is a major concern. Most physicians and specialists are concentrated in urban centers, leaving approximately 65% of the rural population with inadequate access to trained healthcare providers [25]. Low salaries in a competitive market, lack of basic amenities, and non-conducive work environment in rural areas are major deterrents to serving these communities [26]. This situation may partly reflect weaknesses in governance in the public sector, with no clear roadmap for plugging these gaps in human resources for health.

### Engaging stakeholders

More effective and equitable health systems require stakeholders’ engagement so that diverse perspectives and needs are considered in policymaking and implementation [27]. Pakistan’s progress toward UHC will necessitate addressing fears and reservations of the stakeholders by improving governance and ensuring inclusive decision-making. UHC is a broader agenda, and therefore, it requires meaningful coordination between the federal and provincial authorities [6]. Furthermore, within a province, the departments of health, planning, and finance often work in silos, for which an interdepartmental working relationship would be instrumental. Greater coordination between institutions such as the Pakistan Medical & Dental Council, the Nursing and Allied Health Council, healthcare commissions, the Drug Regulatory Authority, and others would likely strengthen implementation. The literature recommends engaging the private health sector and pharmaceutical companies for the regulation of prices of essential medicines and diagnostics. It would be imperative that NGOs and development partners also align their country programs and field projects with the national health agenda [28].

## Population Level

### Low awareness

People residing in the rural areas and belonging to marginalized communities do not have enough awareness about preventive measures such as vaccinations and regular medical check-ups [29]. Moreover, the overall cognizance of UHC in the larger population is very low, particularly information about what the government is doing to achieve the goals of UHC. Socioeconomic disparities result in inequitable access to essential health services [30]. Improved awareness campaigns may improve program uptake and will help in UHC program expansion in order to benefit vulnerable segments of the population [18].

### Health insurance literacy

For providing financial security and ensuring equitable access to quality healthcare services for all citizens, a well-structured health insurance program would be imperative, moving the country closer to the goal of UHC. Such programs have shown to address the disparities in healthcare access and affordability, benefiting the most vulnerable and underserved populations [31]. However, the present situation in Pakistan shows that poor segments who are enrolled in the health insurance program are not well informed about their entitlement [32]. To address this challenge, a potential strategy would be needed involving schools, local media, and community-based organizations to improve health insurance literacy, leading to timely healthcare-seeking.

### Utilization of services

With only half of its population having access to essential healthcare, and that too varies significantly across provinces, Pakistan will face a huge challenge in achieving UHC by 2030. This alludes to the need for political commitment and very focused interventions [18]. Increasing poverty, dearth of responsiveness of public sector, and poor families eventually resorting to private doctors end up incurring catastrophic health expenditure [3]. Service utilization has been low in the health insurance program also because people do not know the range of services that can be availed, the benefits offered, and distrust on the services of empaneled hospitals, particularly those in the government domain [33]. The evidence supports the consideration of expanding poverty reduction strategies for the marginalized population groups and scaling up the health insurance program in Pakistan.

## Service Delivery and Provider Level

### Quality of care

Healthcare-seeking in public sector hospitals is low because of the dismal quality and overall weak responsiveness of the system [4]. Improving quality of care is widely recognized as an important component of UHC [34]. Service quality is no doubt superior in the private sector compared to the public sector, with shorter waiting times, better hospitality, and increased time spent with providers [22, 35]. Public sector hospitals ought to improve their overall quality of care to ensure better health outcomes, rebuild trust among the people served, and maximize resource efficiency. Without improvements in quality, progress toward UHC is likely to remain constrained. For Pakistan, it is utmost important to strengthen its hospitals’ infrastructure, build the skill set of workforce, and institute a robust monitoring system in order to deliver reliable, high-quality care across all levels of health services.

### Empanelment standards

The current standards of many empaneled hospitals show systemic inefficiencies and gaps, and in some cases, there are issues of compliance due to financial barriers. This state of affairs results in inconsistencies in healthcare quality [35]. The Pakistan Medical & Dental Council, healthcare commissions, and provincial health authorities should follow uniform and strict enforcement of quality benchmarks for empanelment of the hospitals in the health insurance program [36, 37]. Due to weak scrutiny, many private hospitals have been empaneled, which actually lack compliance with international accreditation such as ISO or Joint Commission of International Accreditation (JCIA). Moreover, post-empanelment audits should be robust to check any substandard care provided. It is understandable that smaller hospitals and clinics in remote areas will not be able to meet the empanelment requirements due to their resource constraints [38]. A phased national accreditation system could be considered. Moreover, advocacy with the government should be around investing in infrastructure, technology, and staff training in the public hospitals to meet empanelment standards.

### Treatment protocols

Treatment protocols are important for regimenting care and for ensuring dependable, high-quality, and cost-effective health services. These guidelines and protocols guide healthcare providers and help them minimize errors and optimize the use of resources [39]. Such modalities also enhance equity by ensuring that all patients receive the same level of care [40]. Additionally, they improve efficiency, allowing health systems to manage limited resources effectively [14].

### Communication

Pakistan’s healthcare system is highly fragmented, with public and private sectors operating independently. There is weak coordination and communication between federal and provincial health departments [41]. The lack of an integrated health information system may hamper the tracking of healthcare outcomes and the making of evidence-based policies. Data collection mechanisms are outdated, and there is no centralized digital database to record patient information, which leads to insufficient and inefficient resource allocation [42]. The private health sector has numerous reservations about sharing patients’ data, and addressing these concerns may facilitate more comprehensive health information on the state of health of the nation [22].

## Public Health Policy Implications

On the roadmap to UHC, Pakistan must rethink a comprehensive, multi-sectoral strategy with clear priorities. Increasing healthcare spending is essential to strengthen public health infrastructure, services, and resources. Subsequently, the country needs to reform its primary healthcare services to ensure the provision of accessible and equitable care to all citizens. The health insurance program is showing some promising results; it must be scaled up in all provinces. Workforce development is another area that calls for addressing the shortage and skill imbalance. An integrated national health information system is another gap that needs to be plugged by streamlining data sharing, management, and analytics for supporting evidence-based decision-making. Finally, robust public awareness campaigns should be launched with key messages to encourage timely healthcare-seeking. Pakistan can make significant strides toward improving its healthcare system and achieving better health outcomes for its population, which is the philosophy of UHC and eventually country’s socioeconomic development.

## Data Availability

The author had full access to all data and is solely responsible for the content of the manuscript.
